# Person-centred maternity care in low-income and middle-income countries: analysis of data from Kenya, Ghana, and India

**DOI:** 10.1016/S2214-109X(18)30403-0

**Published:** 2018-12-13

**Authors:** Patience A Afulani, Beth Phillips, Raymond A Aborigo, Cheryl A Moyer

**Affiliations:** a Department of Epidemiology and Biostatistics, University of California, San Francisco, CA, USA; b Institute for Global Health Sciences, University of California, San Francisco, CA, USA; c Population and Reproductive Health unit, Navrongo Health Research Centre, Navrongo, Ghana; d Department of Learning Health Sciences and Department of Obstetrics and Gynecology, University of Michigan, Ann Arbor, MI, USA

## Abstract

**Background:**

Several qualitative studies have described disrespectful, abusive, and neglectful treatment of women during facility-based childbirth, but few studies document the extent of person-centred maternity care (PCMC)—ie, responsive and respectful maternity care—in low-income and middle-income countries. In this Article, we present descriptive statistics on PCMC in four settings across three low-income and middle-income countries, and we examine key factors associated with PCMC in each setting.

**Methods:**

We examined data from four cross-sectional surveys with 3625 women aged 15–49 years who had recently given birth in Kenya, Ghana, and India (surveys were done from August, 2016, to October, 2017). The Kenya data were collected from a rural county (n=877) and from seven health facilities in two urban counties (n=530); the Ghana data were from five rural health facilities in the northern region (n=200); and the India data were from 40 health facilities in Uttar Pradesh (n=2018). The PCMC measure used was a previously validated scale with subscales for dignity and respect, communication and autonomy, and supportive care. We analysed the data using descriptive statistics and bivariate and multivariate regressions to examine predictors of PCMC.

**Findings:**

The highest mean PCMC score was found in urban Kenya (60·2 [SD 12·3] out of 90), and the lowest in rural Ghana (46·5 [6·9]). Across sites, the lowest scores were in communication and autonomy (from 8·3 [3.3] out of 27 in Ghana to 15·1 [5·9] in urban Kenya). 3280 (90%) of the total 3625 women across all countries reported that providers never introduced themselves, and 2076 (57%) women (1475 [73%] of 1980 in India) reported providers never asked permission before performing medical procedures. 120 (60%) of 200 women in Ghana and 1393 (69%) of 1980 women in India reported that providers did not explain the purpose of examinations or procedures, and 116 (58%) women in Ghana and 1162 (58%) in India reported they did not receive explanations on medications they were given; additionally, 104 (52%) women in Ghana did not feel able to ask questions. Overall, 576 (16%) women across all countries reported verbal abuse, and 108 (3%) reported physical abuse. PCMC varied by socioeconomic status and type of facility in three settings (ie, rural and urban Kenya, and India).

**Interpretation:**

Regardless of the setting, women are not getting adequate PCMC. Efforts are needed to improve the quality of facility-based maternity care.

**Funding:**

Bill & Melinda Gates Foundation, Marc and Lynne Benioff, and USAID Systems for Health.

## Introduction

Globally, about 300 000 women died from pregnancy-related and childbirth-related causes in 2015. Almost all of these deaths occurred in low-income and middle-income countries (LMICs).^1^ Care by skilled providers is critical to preventing these deaths, yet only about half of births in LMICs occur in health facilities.^2^ Poor person-centred maternity care (PCMC) is a key factor driving both the low proportions of facility-based deliveries and high maternal mortality.^3^, ^4^, ^5^, ^6^, ^7^, ^8^ PCMC refers to maternity care that is respectful of and responsive to individual women and their families' preferences, needs, and values.^9^, ^10^ PCMC thus includes system and provider responsiveness, patient–provider communication, and interpersonal treatment.^7^, ^11^, ^12^ PCMC broadens the growing interests in mistreatment or disrespect and abuse of women during facility-based childbirth, and it highlights respectful maternity care as part of the broader interest in person-centred (patient-centred, people-centred, or woman-centred) care.^13^, ^14^, ^15^, ^16^, ^17^, ^18^ Person-centred care is also a key dimension of quality of care,^9^ which captures the interpersonal dimensions of care described by Donabedian as the means by which care is delivered.^19^

The key domains of PCMC—dignity and respect, communication and autonomy, and supportive care^10^—are represented as the experience-of-care dimensions in the WHO quality-of-care framework for maternal and newborn health, and are highlighted in WHO recommendations for a positive childbirth experience.^20^, ^21^ Poor PCMC, which is characterised by disrespectful and neglectful treatment of women during facility deliveries, has multiplicative effects, as it can deter women from giving birth in health facilities and lead to poor pregnancy outcomes.^7^, ^13^, ^22^, ^23^ Conversely, PCMC can contribute to the timely provision of care, improved patient–provider communication, and increased adherence to treatments, all of which can improve maternal and neonatal outcomes.^7^, ^8^, ^11^, ^12^ Although several qualitative studies have documented disrespectful, abusive, and neglectful treatment of women in facilities during childbirth, there is a paucity of quantitative studies on the extent of PCMC in LMICs.^13^, ^15^

**Research in context**

**Evidence before this study**

We reviewed publications in English on topics related to person-centred maternity care to situate this research in context. We did a basic scientific-literature review of the available evidence searching PubMed for the terms “maternity care” or “maternal health” AND “disrespect”, “abuse”, “mistreatment”, “respectful care”, “dignity”, “quality”, and “patient centered care” OR “person-centered care”. We reviewed research published in the last 20 years (from Jan 1, 1998, to Jan 1, 2018), drawing particularly on systematic reviews of disrespect and abuse and respectful maternity care. We did not assess quality of studies. Previous studies on women's experiences during childbirth have focused on disrespect and abuse, with most studies having a qualitative approach. The growing number of quantitative studies on disrespect and abuse have documented a wide range of estimates on the prevalence of disrespect and abuse, due to different methodological approaches including different operational definitions of disrespect and abuse; different ways of generating summary measures; variations in eligibility criteria; different sampling techniques; and the mode, timing, and setting of data collection. For example, some studies have reported a 15% prevalence of disrespect and abuse, whereas others report a prevalence greater than 90%. These studies also report summary prevalence measures of disrespect and abuse in different ways, with the most common method being a tally of the number of women who answered “yes” to experiencing at least one of the disrespect and abuse dimensions studied. The dimensions most commonly used include physical abuse, non-consented care, non-confidential care, non-dignified care, discrimination, abandonment of care, and detention in facilities, based on Bowser and Hill's landscape analysis. The exact operationalisation of these dimensions differs across studies, making comparison difficult. For example, the prevalence of non-consented care measured in different ways across studies in sub-Saharan Africa have ranged from less than 1% in some studies to over 20% in others. Reviews of key methodological, conceptual, and operational components of disrespect and abuse and respectful maternity care in facility-based childbirths have called for further research to standardize these constructs and improve the validity and reliability of measures. There is thus much work to be done in measuring women's childbirth experiences in various settings.

**Added value of this study**

This study extends the literature on women's experiences during childbirth by focusing on the broader construct of person-centred maternity care (PCMC), applying a specific operationalisation of PCMC, and measuring PCMC with the same validated PCMC tool in four settings across three low-income and middle-income countries. The consistent operationalisation of PCMC enables us to present a cross-sectional view of PCMC in these settings. This Article is among the few studies to quantitatively measure PCMC in poor-resource settings and, to the best of our knowledge, it is the first study to use the same validated PCMC tool across three low-income and middle-income countries in two continents, thus enabling a comparison of PCMC indicators across the settings. We present both summative PCMC scores and individual indicators of PCMC to highlight areas that are lacking across the four settings to help inform quality-improvement targets for PCMC. We also present patient and facility characteristics associated with PCMC.

**Implications of all the available evidence**

This study shows that across four different study settings in three countries in sub-Saharan Africa and south Asia, women are not receiving PCMC. Communication and respect for women's autonomy tend to be poor across all our study sites, highlighting a key gap in PCMC. PCMC differs by women's socioeconomic status and the level of facility they deliver in, and gaps exist in the provision of dignified and supportive care. This study indicates that much work is needed to improve the quality of maternity care that women receive when they deliver in health-care facilities throughout the developing world. It also highlights the PCMC dimensions where more work is needed, both across the four settings and within each, thus presenting potential opportunities for specific quality-improvement initiatives to address these gaps.

Quantitative studies on women's experience of care during childbirth have focused on measuring the extent of disrespect and abuse. These studies, however, document a wide range of estimates, partly due to different methodological approaches, including varying operational definitions of the construct of interest and non-standardised generation of summary measures.^24^ For example, a Kenya study^25^ found a 20% prevalence of disrespect and abuse based on a single question with a yes or no response, asking women whether at any point during labour and delivery they were treated in a way that made them feel humiliated or disrespected. Other studies^26^, ^27^, ^28^, ^29^, ^30^ reported prevalence of disrespect and abuse by tallying the number of women who answered affirmatively to experiencing at least one of the disrespect and abuse dimensions from Bowser and Hill's landscape analysis.^31^ These studies have found prevalence of disrespect and abuse to range from 15% in Tanzania (with 70% in a follow-up survey),^26^ to 21% and 77% in India (based on different measures),^27^, ^28^ to over 90% in Nigeria and Peru.^29^, ^30^ Qualitative studies in Ghana have documented evidence of poor PCMC,^22^, ^23^, ^32^ but, to our knowledge, none has examined this observation quantitatively.

In this Article, we seek to further research on quality of maternity care and to address these measurement and estimation gaps by focusing on the broader construct of PCMC and operationalising the construct consistently across settings using a validated tool—the PCMC scale—which has been shown to be valid and reliable in LMICs.^10^, ^33^ We present descriptive statistics on PCMC in four settings across three LMICs where the same PCMC tool was used, to highlight specific PCMC aspects that are lacking across these settings. We also examine key factors associated with PCMC in each setting. On the basis of previous research, we hypothesise that PCMC will be suboptimal across all settings, communication and autonomy will have the lowest scores across settings, and PCMC will differ by client and facility characteristics in each setting.

## Methods

### Data sources

The data for this analysis are from four different surveys: two in Kenya^10^ and one each in Ghana (unpublished) and India.^33^ These were independent studies with different study goals, in-country collaborating partners, data-collection teams, and study procedures. The University of California, San Francisco (UCSF), USA, was, however, a common collaborating partner across all four studies. Additionally, the questionnaire for each of the surveys included the PCMC scale and some questions on characteristics of the respondents and the facility they gave birth in. Respondents in all studies were postpartum women and girls aged 15–49 years and all gave written informed consent after receiving information about the research. Differences in the four data sources are summarised in table 1.

**Table 1:** Data sources

	**Rural Kenya**	**Urban Kenya**	**Ghana**	**India**
Location	Migori County	Nairobi and Kiambu Counties	East Mamprusi district	20 districts in Uttar Pradesh
Type of setting	Rural	Predominantly urban	Rural	Predominantly rural
Data collection period	August–September, 2016	August–December, 2016	March–April, 2017	August–October, 2017
Purpose of data collection	Research study on quality of maternity care	Baseline data for a quality-improvement intervention	Baseline data for a quality-improvement intervention	Research study on quality of maternity care
Recruitment sites	20 public and private health facilities across the county and in the homes of women	7 public health facilities	4 public and 1 private health facility	40 public health facilities
Interview location	Health facilities and respondents' homes	Health facilities	Health facilities and respondents' homes	Health facilities
Time of interview after delivery	Within 9 weeks of delivery	Within 1 week of delivery	Within 8 weeks of delivery	Within 48 h of delivery
Interview language	English, Swahili, and Luo	English and Swahili	Mampruli and Kokomba	Hindi
Collaborating partners	UCSF and KEMRI	UCSF and IPA	UCSF and NHRC	UCSF and CEL
Interviewers	Field staff hired by UCSF global programmes office in Kenya	Field staff hired by IPA	Field staff hired by NHRC	Field staff hired by CEL
Ethics approval	UCSF and KEMRI ethical-review units	UCSF and KEMRI ethical-review units	UCSF and NHRC ethical-review units	UCSF and CEL ethical-review units

UCSF=University of California, San Francisco. KEMRI=Kenya Medical Research Institute (Kenya). IPA=Innovations for Poverty Action (Kenya). NHRC=Navrongo Health Research Center (Ghana). CEL=Community Empowerment Lab (India).

The proposal and study materials for the projects that provide data for this manuscript were reviewed and approved by the UCSF Committee for Human Subjects, the Kenya Medical Research Institute Scientific and Ethics Review Unit, the Navrongo Health Research Center in Ghana, and the Community Empowerment Lab in India.

In Kenya, one survey was done in the Migori County—a predominantly rural county in western Kenya. The survey was part of a research study on community perceptions of quality of care during childbirth.^10^, ^34^, ^35^, ^36^ The interviews were in English, Swahili, and Luo and took place in private spaces at health facilities or in the homes of the respondents. Data were collected by use of the REDCap application on a tablet, with data uploaded directly online. 1052 women were interviewed, with 433 (41%) of the interviews held at a health facility. We analysed data from women who delivered in a health facility (n=894) and who provided complete information on the PCMC items (n=877). We refer to this sample as the rural Kenya group.

The other survey in Kenya was done at seven government health facilities in the Nairobi and Kiambu Counties. Nairobi is the national capital of Kenya and is 100% urban. The Kiambu County, which is adjacent to Nairobi, is 60% urban, but the study facilities were located in the urban portions of the county.^37^ The survey was done to obtain baseline data for the evaluation of a project for the improvement of person-centred care quality. Interviews were in English or Swahili, or both, and took place in a private space at the facility. Data from the interviews were collected using the SurveyCTO platform on a tablet, with data uploaded to the server at the end of each day. 531 women were interviewed. We analysed data from women who provided complete information on all the PCMC items (n=530). We refer to this sample as the urban Kenya group. The sampling procedures for the two Kenya surveys are described in detail elsewhere.^10^

In Ghana, the survey was conducted in five health facilities in the East Mamprusi district—a rural district in northern Ghana. The survey was done to obtain baseline data for the evaluation of an intervention for the improvement of maternal and newborn quality of care. The interviews were held in Mampruli and Kokomba, in private spaces at the health facilities and in the women's homes. Interviews were all paper based, and responses were subsequently entered into the REDCap portal on a computer. 268 women were interviewed. We analysed data from women who delivered in a health facility (n=227) and who provided complete information on the PCMC variables (n=200). We refer to this sample as the Ghana group.

In India, the survey was conducted in 40 public health facilities in 20 predominantly rural districts of Uttar Pradesh, a state in northern India. The survey was done as part of a cross-sectional study on quality of maternity care in Uttar Pradesh. All interviews were in Hindi and took place at the health facility, most of them (2015 of 2018 interviews) in the postnatal ward at the patient's bed. Interviews were held using the CommCare platform on tablets, with data uploaded to the server at the end of each day. 2018 women were interviewed, with roughly 50 women interviewed per facility. We refer to this sample as the India group.

### Dependent-variable measure: PCMC scale

We measured PCMC on the PCMC scale, which was initially validated in the Kenya group and subsequently in the India group, and shown to have high content, construct, and criterion validity and to offer good internal-consistency reliability (described in detail elsewhere).^10^, ^33^ The scale includes 30 items that span three domains: dignity and respect, communication and autonomy, and supportive care. Each item has a four-point response scale—ie, 0 (“no, never”), 1 (“yes, a few times”), 2 (“yes, most of the time”), and 3 (“yes, all the time”). The process towards the development of the scale included literature and expert reviews to assess content validity, cognitive interviews with women to evaluate wording and appropriateness of the items, and psychometric analysis using survey data to assess construct and criterion validity and reliability. The validation of the PCMC scale was one of the objectives of the Kenya studies and that of a related study in India. The final scale is based on findings from expert reviews and cognitive interviews from both Kenya and India, with iterative translation from English to the local languages at each stage.^10^ This scale was used in Ghana with only minor modifications during pretesting. The full scale and subscales have good internal-consistency reliability in all the groups, with a Cronbach's α value of over 0·8 for the full scale across all groups and ranging between 0·61 and 0·75 for the subscales. The overall PCMC score is a summative score from the responses to individual items in the 30-item PCMC scale (with negative items reverse coded—ie, questions that were framed negatively, such as the physical and verbal abuse questions, had to be recoded so that high numbers represent good care). The minimum possible score is 0 and the maximum possible is 90, with a low score indicating poor PCMC. In addition to presenting overall PCMC scores and domain scores, we examined individual items to highlight gaps in key dimensions of PCMC.

### Independent-variable measures

We examined potential predictors of PCMC using variables that were captured similarly in all four groups. These included demographic variables such as age, parity, and marital status and measures of socioeconomic status (ie, education, employment, and household wealth). We also included variables to capture complications, antenatal attendance, and facility and provider characteristics. Facilities were characterised by the type of facility the woman delivered in, categorised as public or government hospital (high level), health centre (low level), or private or mission health facility (too few to group by levels). Provider characteristics were type (ie, skilled providers, including nurses or midwifes, clinical officers or medical assistants, and doctors; non-skilled providers, including support staff or traditional birth attendants; and more than one skilled provider) and gender of delivery providers.

### Statistical analysis

All data were retrieved from REDCap, SurveyCTO, and CommCare and imported into Stata 15 for cleaning and analysis. Analysis involved descriptive statistics for each of the groups and bivariate and multivariate analyses to examine associations between independent variables and the PCMC score. We first examined mean differences in PCMC by all the potential predictors using cross tabulations and unadjusted ordinary least-squares regressions, as the PCMC scale is normally distributed. We then built the multivariate models for each group by including all variables that were significantly associated with PCMC in the bivariate models for at least one group. We also included variables such as age, parity, and pregnancy complications that were not significant in any of our bivariate models, but which we believed were potential predictors of PCMC on the basis of previous research.^13^, ^25^, ^27^, ^38^ A p value of less than 0·05 was considered significant. We did not combine the datasets to test statistical significance between countries because of the described differences between groups.

### Role of the funding source

The sponsors of the study had no role in the study design, data collection, data analysis, data interpretation, or writing of the Article. The corresponding author had full access to all of the data in the study and had final responsibility for the decision to submit for publication.

## Results

Table 2 shows the demographic characteristics of respondents for all the groups. The average age was approximately 25 years for the Kenya and India groups and 30 years for the Ghana group. The average parity was three children for the rural Kenya and the Ghana groups, and about two children for the urban Kenya group and the India group. More than 90% of the women in the Ghana and India groups were married compared with a little over 70% in Kenya. Women in the Ghana group had received the least education and were the most likely to be unemployed and in the lowest wealth quintile.

**Table 2:** Demographic characteristics of respondents and potential predictors of PCMC for all groups

		**Rural Kenya**	**Urban Kenya**	**Ghana**	**India**
Total number in group*		877 (100%)	530 (100%)	200 (100%)	2018 (100%)
Age, n (mean years [SD])		857 (25·0 [5·9])	530 (25·6 [4·8])	199 (29·5 [6·7])	2018 (25 [4·0])
Parity, n (mean years [SD])		856 (2·8 [2·0])	530 (2·1 [1·1])	197 (3·3 [1·8])	2018 (2·2 [1·3])
Marital status					
	Single	140 (16%)	61 (12%)	4 (2%)	0 (0%)
	Partnered or cohabiting	3 (0%)	75 (14%)	8 (4%)	0 (0%)
	Married	687 (78%)	382 (72%)	188 (94%)	2013 (100%)
	Widowed	35 (4%)	1 (0%)	0 (0%)	2 (0%)
	Divorced or separated	12 (1%)	11 (2%)	0 (0%)	3 (0%)
	Total	877	530	200	2018
Education					
	No school or primary	495 (56%)	204 (39%)	149 (75%)	941 (47%)
	Postprimary, vocational, or secondary	271 (31%)	241 (46%)	48 (24%)	828 (41%)
	College or above	111 (13%)	85 (16%)	3 (12%)	249 (12%)
	Total	877	530	200	2018
Employed					
	No	658 (75%)	251 (47%)	178 (89%)	1905 (94%)
	Yes	219 (25%)	279 (53%)	21 (11%)	113 (6%)
	Total	877	530	199	2018
Wealth quintile†					
	Poorest	190 (22%)	0 (0%)	57 (30%)	404 (20%)
	Poor	190 (22%)	1 (0%)	53 (28%)	404 (20%)
	Middle	135 (15%)	18 (3%)	77 (40%)	403 (20%)
	Rich	172 (20%)	88 (17%)	3 (2%)	404 (20%)
	Richest	190 (22%)	423 (80%)	1 (1%)	403 (20%)
	Total	877	530	191	2018
Religion					
	Christian	862 (98%)	523 (99%)	49 (25%)	1 (0%)
	Muslim or other	15 (2%)	7 (1%)	149 (75%)	342 (17%)
	Hindu	0 (0%)	0 (0%)	0 (0%)	1675 (83%)
	Total	877	530	198	2018
Pregnancy complications					
	No	494 (56%)	446 (84%)	102 (51%)	425 (21%)
	Yes	383 (44%)	84 (16%)	98 (49%)	1593 (79%)
	Total	877	530	200	2018
Number of antenatal care visits					
	No antenatal care	6 (1%)	225 (43%)	2 (1%)	205 (10%)
	<4	281 (32%)	247 (47%)	13 (7%)	1466 (73%)
	≥4	585 (67%)	53 (10%)	183 (92%)	347 (17%)
	Total	872	525	198	2018
Delivery facility type					
	Government hospital	404 (46%)	431 (81%)	22 (11%)	703 (35%)
	Government health centre	362 (41%)	99 (19%)	85 (43%)	1315 (65%)
	Mission or private facility	111 (13%)	0 (0%)	92 (46%)	0 (0%)
	Total	877	530	199	2018
Delivery provider					
	Nurse or midwife	656 (75%)	268 (51%)	171 (86%)	1717 (85%)
	Doctor	83 (10%)	146 (28%)	12 (6%)	44 (2%)
	Clinical officer or medical assistant	54 (6%)	1 (0%)	5 (3%)	18 (1%)
	Non-skilled attendant	21 (2%)	3 (1%)	9 (5%)	239 (12%)
	>1 skilled providers	63 (7%)	112 (21%)	3 (2%)	0 (0%)
	Total	877	530	200	2018
Delivery provider gender					
	Male	329 (38%)	73 (14%)	8 (4%)	9 (1%)
	Female	514 (59%)	371 (70%)	189 (95%)	2001 (100%)
	Both	34 (4%)	86 (16%)	2 (1%)	0 (0%)
	Total	877	530	199	2010

Data are n (%) unless otherwise specified in row heading. Percentages might not add up to 100 because of rounding. PCMC=person-centred maternity care.

* The total sample in the first row is the initial analytic sample representing women with complete data on the PCMC variables. The proportion of missing variables in each sample on the PCMC variable is 1·9% for rural Kenya, 0·2% for urban Kenya, 11·9% for Ghana, and 0% for India. However, there were missing data on some of the predictors indicated by variable totals less than the sample total at the top of the column. Missing observations on predictors included in the multivariate models account for the smaller analytic sample for the multivariate analysis.

† Household wealth is measured in quintiles calculated from a wealth index based on several questions on household assets in each of the datasets. The wealth indices in Kenya and Ghana are weighted to be relative to wealth levels in each country using procedures described in the equity tool kit.^20^ Wealth indices in the India group are not weighted because of differences in the asset questions asked.

Table 3 shows the distribution of all the PCMC variables by domain. Several items in the different domains pointed to poor PCMC across all the countries.

**Table 3:** Distribution of person-centred maternity care variables by country and setting

		**Rural Kenya (n=877)**	**Urban Kenya (n=530)**	**Ghana (n=200)**	**India (n=2018)**
**Items under dignity and respect subscale**					
Did the doctors, nurses, or other staff at the facility treat you with respect?					
	0 No, never	21 (2%)	11 (2%)	12 (6%)	143 (7%)
	1 Yes, a few times	78 (9%)	65 (12%)	67 (34%)	299 (15%)
	2 Yes, most of the time	254 (29%)	151 (28%)	97 (49%)	531 (26%)
	3 Yes, all the time	524 (60%)	303 (57%)	24 (12%)	1045 (52%)
Did the doctors, nurses, and other staff at the facility treat you in a friendly manner?					
	0 No, never	29 (3%)	23 (4%)	10 (5%)	92 (5%)
	1 Yes, a few times	99 (11%)	66 (12%)	68 (34%)	358 (18%)
	2 Yes, most of the time	242 (28%)	187 (35%)	104 (52%)	545 (27%)
	3 Yes, all the time	506 (58%)	254 (48%)	18 (9%)	1023 (51%)
Did you feel the doctors, nurses, or other health-care providers shouted at you, scolded, insulted, threatened, or talked to you rudely?					
	0 No, never	778 (89%)	435 (82%)	175 (88%)	1661 (82%)
	1 Yes, once	58 (7%)	0 (0%)	15 (8%)	212 (11%)
	2 Yes, a few times	24 (3%)	85 (16%)	6 (3%)	131 (6%)
	3 Yes, many times	17 (2%)	10 (2%)	4 (2%)	14 (1%)
Did you feel like you were treated roughly like pushed, beaten, slapped, pinched, physically restrained, or gagged?					
	0 No, never	838 (96%)	520 (98%)	192 (96%)	1967 (97%)
	1 Yes, once	24 (3%)	0 (0%)	6 (3%)	31 (2%)
	2 Yes, a few times	10 (1%)	10 (1%)	2 (1%)	17 (1%)
	3 Yes, many times	5 (0%)	0 (0%)	0 (0%)	3 (0%)
During examinations in the labour room, were you covered up?					
	0 No, never	178 (20%)	204 (38%)	7 (4%)	526 (26%)
	1 Yes, a few times	64 (7%)	46 (9%)	14 (7%)	115 (6%)
	2 Yes, most of the time	114 (13%)	55 (10%)	61 (31%)	228 (11%)
	3 Yes, all the time	512 (58%)	199 (38%)	118 (59%)	1149 (57%)
	4 Not applicable	9 (1%)	26 (5%)	0 (0%)	0 (0%)
Do you feel like your health information was or will be kept confidential at this facility?					
	0 No, never	55 (6%)	21 (4%)	8 (4%)	324 (16%)
	1 Yes, a few times	110 (13%)	58 (11%)	49 (25%)	444 (22%)
	2 Yes, most of the time	268 (31%)	132 (25%)	61 (31%)	387 (19%)
	3 Yes, all the time	444 (51%)	319 (60%)	82 (41%)	863 (43%)
**Items under Communication and Autonomy subscale**					
During your time in the health facility did the doctors, nurses, or other health-care providers introduce themselves to you when they first came to see you?					
	0 No, none of them	675 (77%)	451 (85%)	174 (87%)	1980 (98%)
	1 Yes, a few of them	108 (12%)	42 (8%)	9 (5%)	35 (2%)
	2 Yes, most of them	41 (5%)	27 (5%)	16 (8%)	2 (0%)
	3 Yes, all of them	53 (6%)	10 (2%)	1 (1%)	1 (0%)
Did the doctors, nurses, or other health-care providers call you by your name?					
	0 No, never	236 (27%)	231 (44%)	86 (43%)	567 (28%)
	1 Yes, a few times	177 (20%)	131 (25%)	30 (15%)	436 (22%)
	2 Yes, most of the time	136 (16%)	94 (28%)	17 (9%)	371 (18%)
	3 Yes, all the time	328 (37%)	74 (14%)	67 (34%)	644 (32%)
Did you feel like the doctors, nurses or other staff at the facility involved you in decisions about your care?					
	0 No, never	171 (20%)	43 (8%)	83 (42%)	1131 (56%)
	1 Yes, a few times	117 (13%)	48 (9%)	69 (35%)	311 (15%)
	2 Yes, most of the time	172 (20%)	66 (12%)	33 (17%)	255 (13%)
	3 Yes, all the time	345 (39%)	236 (45%)	14 (7%)	321 (16%)
	4 Did not have to make any decisions	71 (8%)	137 (26%)	1 (1%)	0 (0%)
During the delivery, do you feel like you were able to be in the position of your choice?					
	0 No, never	614 (70%)	209 (39%)	118 (59%)	360 (18%)
	1 Yes, for a short time	110 (13%)	46 (9%)	36 (18%)	655 (32%)
	2 Yes, most of the time	74 (8%)	86 (16%)	44 (22%)	418 (21%)
	3 Yes, all the time	79 (9%)	189 (36%)	2 (1%)	585 (29%)
Did the doctors, nurses, or other staff at the facility speak to you in a language you could understand?					
	0 No, never	22 (3%)	1 (0%)	11 (6%)	16 (0%)
	1 Yes, a few times	69 (8%)	7 (1%)	27 (14%)	131 (6%)
	2 Yes, most of the time	190 (22%)	58 (11%)	80 (40%)	315 (16%)
	3 Yes, all the time	596 (68%)	464 (88%)	82 (41%)	1556 (77%)
Did the doctors, nurses, or other staff at the facility ask your permission or consent before doing procedures on you?					
	0 No, never	318 (36%)	196 (37%)	87 (44%)	1475 (73%)
	1 Yes, a few times	119 (14%)	80 (15%)	53 (27%)	282 (14%)
	2 Yes, most of the time	200 (23%)	130 (25%)	43 (22%)	172 (9%)
	3 Yes, all the time	240 (27%)	124 (23%)	17 (9%)	89 (4%)
Did the doctors and nurses explain to you why they were doing examinations or procedures on you?					
	0 No, never	244 (28%)	107 (20%)	120 (60%)	1393 (69%)
	1 Yes, a few times	123 (14%)	82 (15%)	39 (20%)	344 (17%)
	2 Yes, most of the time	219 (25%)	127 (23%)	33 (17%)	174 (9%)
	3 Yes, all the time	291 (33%)	214 (40%)	8 (4%)	107 (5%)
Did the doctors and nurses explain to you why they were giving you any medicine?					
	0 No, never	153 (17%)	94 (18%)	116 (58%)	1162 (58%)
	1 Yes, a few times	108 (12%)	68 (13%)	48 (24%)	400 (20%)
	2 Yes, most of the time	212 (24%)	58 (11%)	29 (15%)	242 (12%)
	3 Yes, all the time	329 (38%)	166 (31%)	6 (3%)	205 (10%)
	4 Did not get any medicine	73 (8%)	144 (27%)	1 (1%)	9 (0%)
Did you feel you could ask the doctors, nurses, or other staff at the facility any questions you had?					
	0 No, never	201 (23%)	101 (19%)	104 (52%)	265 (13%)
	1 Yes, a few times	211 (24%)	91 (17%)	44 (22%)	437 (22%)
	2 Yes, most of the time	185 (21%)	159 (30%)	47 (24%)	543 (27%)
	3 Yes, all the time	280 (32%)	179 (34%)	5 (3%)	773 (38%)
**Items under Supportive Care subscale**					
How did you feel about the amount of time you waited? Would you say it was					
	0 Very short	533 (61%)	250 (47%)	86 (43%)	1347 (67%)
	1 Somewhat short	202 (23%)	141 (27%)	81 (41%)	410 (20%)
	2 Somewhat long	87 (10%)	43 (8%)	28 (14%)	177 (9%)
	3 Very long	55 (6%)	96 (18%)	5 (3%)	84 (4%)
Did the doctors and nurses at the facility talk to you about how you were feeling?					
	0 No, never	117 (13%)	99 (19%)	74 (37%)	817 (40%)
	1 Yes, a few times	264 (30%)	110 (21%)	86 (43%)	776 (38%)
	2 Yes, most of the time	226 (26%)	193 (36%)	39 (20%)	326 (16%)
	3 Yes, all the time	269 (31%)	128 (24%)	1 (1%)	99 (5%)
Did the doctors, nurses, or other staff at the facility try to understand your anxieties?					
	0 No, never	204 (23%)	130 (25%)	74 (37%)	456 (23%)
	1 Yes, a few times	205 (23%)	59 (11%)	84 (42%)	667 (33%)
	2 Yes, most of the time	151 (17%)	86 (16%)	29 (15%)	442 (22%)
	3 Yes, all the time	184 (21%)	100 (19%)	11 (6%)	453 (22%)
	4 I did not have any anxieties or fears	133 (15%)	155 (29%)	2 (1%)	0 (0%)
When you needed help, did you feel the doctors, nurses, or other staff at the facility paid attention?					
	0 No, never	42 (5%)	27 (5%)	34 (17%)	80 (4%)
	1 Yes, a few times	128 (15%)	99 (19%)	66 (33%)	403 (20%)
	2 Yes, most of the time	330 (38%)	246 (46%)	80 (40%)	634 (31%)
	3 Yes, all the time	377 (43%)	158 (30%)	20 (10%)	901 (45%)
Do you feel the doctors or nurses did everything they could to help control your pain?					
	0 No, never	336 (38%)	219 (41%)	40 (20%)	182 (9%)
	1 Yes, a few times	139 (16%)	80 (15%)	70 (35%)	478 (24%)
	2 Yes, most of the time	189 (22%)	132 (25%)	67 (34%)	759 (38%)
	3 Yes, all the time	213 (24%)	99 (19%)	23 (12%)	599 (30%)
Were you allowed to have someone you wanted (outside of staff at the facility, such as family or friends) to stay with you during labour?					
	0 No, never	165 (19%)	242 (46%)	63 (32%)	157 (8%)
	1 Yes, a few times	110 (13%)	17 (3%)	38 (19%)	92 (5%)
	2 Yes, most of the time	234 (27%)	9 (2%)	34 (17%)	295 (15%)
	3 Yes, all the time	362 (41%)	25 (5%)	64 (32%)	1474 (73%)
	4 I did not want someone to stay with me	4 (0%)	237 (45%)	1 (1%)	0 (0%)
Were you allowed to have someone you wanted to stay with you during delivery?					
	0 No, never	534 (61%)	240 (45%)	140 (70%)	175 (9%)
	1 Yes, a few times	76 (9%)	10 (2%)	15 (8%)	79 (4%)
	2 Yes, most of the time	110 (13%)	2 (0%)	37 (19%)	270 (13%)
	3 Yes, all the time	145 (17%)	20 (4%)	8 (4%)	1494 (74%)
	4 I did not want someone to stay with me	12 (1%)	258 (49%)	0 (0%)	0 (0%)
Did you feel the doctors, nurses, or other staff at the facility took the best care of you?					
	0 No, never	22 (3%)	6 (1%)	5 (3%)	69 (3%)
	1 Yes, a few times	85 (10%)	75 (14%)	42 (21%)	444 (22%)
	2 Yes, most of the time	310 (35%)	239 (45%)	119 (60%)	831 (41%)
	3 Yes, all the time	459 (52%)	210 (40%)	34 (17%)	674 (33%)
Did you feel you could completely trust the doctors, nurses, or other staff at the facility with regards to your care?					
	0 No, never	29 (3%)	11 (2%)	9 (4%)	55 (3%)
	1 Yes, a few times	90 (10%)	60 (11%)	44 (22%)	144 (7%)
	2 Yes, most of the time	300 (34%)	171 (32%)	104 (52%)	453 (22%)
	3 Yes, all the time	458 (52%)	288 (54%)	43 (21%)	1366 (68%)
Do you think there were enough health staff in the facility to care for you?					
	0 No, never	115 (13%)	58 (11%)	17 (8%)	36 (2%)
	1 Yes, a few times	125 (14%)	77 (15%)	107 (53%)	320 (16%)
	2 Yes, most of the time	255 (29%)	198 (37%)	67 (33%)	696 (34%)
	3 Yes, all the time	382 (43%)	197 (37%)	9 (4%)	966 (48%)
Thinking about the labour and postnatal wards, did you feel the health facility was crowded?					
	0 No, never	404 (46%)	285 (54%)	42 (21%)	427 (21%)
	1 Yes, a few times	187 (21%)	51 (10%)	100 (50%)	803 (40%)
	2 Yes, most of the time	143 (16%)	67 (13%)	49 (24%)	563 (28%)
	3 Yes, all the time	141 (16%)	127 (24%)	9 (4%)	225 (11%)
Thinking about the wards, washrooms, and the general environment of the health facility, would you say the facility was very clean, clean, dirty, or very dirty?					
	0 Very dirty	10 (1%)	102 (19%)	0 (0%)	355 (18%)
	1 Dirty	107 (12%)	397 (75%)	23 (11%)	386 (19%)
	2 Clean	614 (70%)	28 (5%)	168 (84%)	118 (6%)
	3 Very clean	146 (17%)	3 (1%)	9 (4%)	1159 (57%)
Was there water in the facility?					
	0 No, never	39 (4%)	7 (1%)	3 (1%)	264 (13%)
	1 Yes, a few times	73 (8%)	33 (6%)	49 (24%)	48 (2%)
	2 Yes, most of the time	227 (26%)	142 (27%)	95 (47%)	222 (11%)
	3 Yes, all the time	538 (61%)	348 (66%)	53 (26%)	1484 (74%)
Was there electricity in the facility?					
	0 No, never	49 (6%)	0 (0%)	0 (0%)	8 (0%)
	1 Yes, a few times	74 (8%)	15 (3%)	24 (12%)	135 (7%)
	2 Yes, most of the time	258 (29%)	80 (15%)	58 (29%)	920 (46%)
	3 Yes, all the time	496 (57%)	435 (82%)	118 (59%)	955 (47%)
In general, did you feel safe in the health facility?					
	0 No, never	15 (1%)	0 (0%)	0 (0%)	30 (1%)
	1 Yes, a few times	59 (6%)	34 (6%)	35 (17%)	45 (2%)
	2 Yes, most of the time	197 (22%)	91 (17%)	91 (45%)	226 (11%)
	3 Yes, all the time	606 (69%)	405 (76%)	74 (37%)	1717 (85%)

Data are n (%).

A little over 50% of women in the Kenya and India groups felt they were treated with respect all the time, compared with only 12% of women in the Ghana group. More than 10% of women in the rural Kenya (11%), urban Kenya (18%), Ghana (12%), and India (18%) groups reported verbal abuse at least once during their time at the facility, but less than 5% across all groups reported any physical abuse. More than a fifth of women in the Kenya and India groups reported being physically exposed all the time (never covered) during examinations.

Across all groups, more than three-quarters of women (almost all [98%] in the India group) reported that providers never introduced themselves. More than a quarter of the women from all the groups reported that providers never called them by their names. Over 40% of women in the Ghana and India groups felt that providers did not involve them in their care, and between 39% and 70% of women in the Kenya and Ghana groups did not feel they could be in a position of their choice during delivery. Additionally, more than a third of respondents across all groups (and up to 70% in India) said providers never asked permission before doing procedures on them. Another substantial proportion of respondents indicated that providers did not explain the purpose of the examinations, procedures, or medications being administered (about 20% in Kenya and more than 50% in Ghana and India).

Nearly 40% of women in the Ghana and India groups reported providers never talked to them about how they were feeling; and between 20% of women in the Ghana group and 40% of women in the Kenya group felt providers did not do their best to control their pain. Many women in the Kenya and Ghana groups were not given continuous support during labour and delivery, with about half of women in the urban Kenya group labouring without a companion and 70% of women in the Ghana group delivering without a companion.

Mean PCMC scores on the full scale and subscales are shown in table 4. The average PCMC scores for the four groups ranged between 46 and 60 (where 0 is the worst score and 90 is the best score), with the lowest score of 46·5 in the Ghana group and the highest score of 60·2 in the urban Kenya group. To enable a comparison across the domains, we show the rescaled scores (ie, scores shown as a fraction of the total possible score on that domain and normalised to 100) in the figure. These results show gaps in PCMC across all the domains in each setting; the area lacking most is communication and autonomy, especially in the Ghana and India groups where the communication score is less than 40% of the maximum possible score.

**Table 4:** Distribution of full PCMC scale and subscales

	**Cronbach's α value**	**Mean raw scores (SD; min–max range)**
**Rural Kenya (n=877)**		
Full PCMC scale*	0·88	59·5 (13·6; 21–90)
Dignity and respect†	0·66	15·1 (2·9; 3–18)
Communication and autonomy‡	0·78	13·9 (5·9; 1–27)
Supportive Care§	0·75	30·5 (6·8; 8–45)
**Urban Kenya (n=530)**		
Full PCMC Scale*	0·83	60·2 (12·3; 22–86)
Dignity and respect†	0·61	14·4 (2·9; 3–18)
Communication and autonomy‡	0·62	15·1 (4.7; 3–26)
Supportive Care§	0·72	30·4 (6·5; 10–44)
**Ghana (n=200)**		
Full PCMC Scale*	0·84	46·5 (6·9; 29–72)
Dignity and respect†	0·62	13·6 (2·5; 3–18)
Communication and autonomy‡	0·72	8·3 (3.3; 1-17)
Supportive Care§	0·66	24·6 (4·0; 16–38)
**India (n=2018)**		
Full PCMC Scale*	0·85	55·8 (11·6; 18–87)
Dignity and respect†	0·70	14·1 (3·5; 2–18)
Communication and autonomy‡	0·67	9·6 (4.3; 0-25)
Supportive Care§	0·71	32·2 (6·0; 12–45)

PCMC=person-centred maternity care.

* Full PCMC scale has 30 items each on a scale of 0 to 3; therefore, the scores range from 0 to 90.

† The dignity and respect subscale has 6 items and scores range from 0 to 18.

‡ The communication and autonomy subscale has 9 items and scores range from 0 to 27.

§ The supportive-care subscale has 15 items and scores range from 0 to 45.

**Figure:**
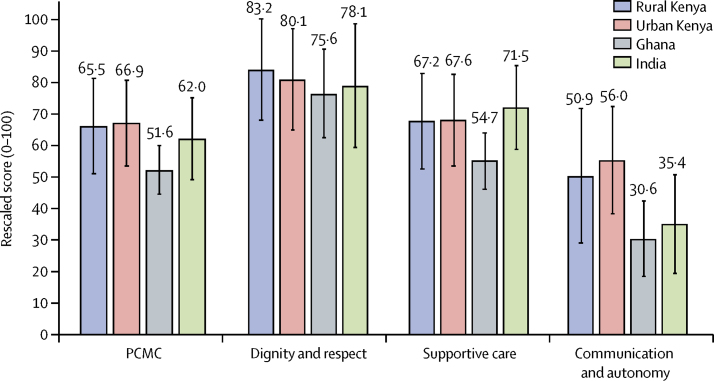
Rescaled scores on person-centred maternity care full scale and subscales Rescaled scores are calculated as the fraction of the total possible score on each domain and normalised to 100. Error bars are rescaled SD values. PCMC=person-centred maternity care.

Table 5 shows the mean PCMC score by selected predictors. In the rural Kenya group, women who were married, college educated, employed, and wealthier reported, on average, a higher PCMC score than did women who were unmarried, less educated, unemployed, and poorer. Additionally, women who delivered in health centres or private facilities, or received care from two providers of different genders reported a higher PCMC score than those who delivered in public hospitals and were assisted by only male or female providers. Finally, women who were interviewed a week or more after delivery and those who were interviewed in their homes reported a lower PCMC score than did those who were interviewed within a week of delivery and in the health facilities. Except for the effect of education, all the significant associations in the bivariate analysis remained significant in the multivariate analysis (table 6). When education was replaced by literacy in the multivariate analysis, literate women report a higher PCMC score than illiterate women (this has been reported elsewhere).^34^

**Table 5:** Bivariate distribution of person-centred-maternity-care scores by key predictors

		**Rural Kenya**		**Urban Kenya**		**Ghana**		**India**	
		Mean (SD)	p value	Mean (SD)	p value	Mean (SD)	p value	Mean (SD)	p value
Total		59·0 (14·0)		60·2 (12·3)		46·5 (6·9)		54·6 (11·2)	
Age									
	15–19 years	57·1 (14·5)	Ref	58·2 (13·0)	Ref	46·1 (6·9)	0·171	53·9 (12·3)	Ref
	20–29 years	59·4 (14·0)	0·068	59·7 (12·1)	0·558	47·3 (7·5)	Ref	55·6 (11·7)	0·210
	30–48 years	59·2 (13·5)	0·164	62·5 (12·5)	0·121	45·1 (5·3)	0·477	56·9 (10·9)	0·047
Marital status									
	Single	56·4 (14·6)	Ref	56·9 (13)	Ref	43·2 (4·1)	Ref	55·8 (11·6)	Ref
	Partnered or cohabiting	55·3 (13·3)	0·897	67·9 (9·6)	<0·0001	45·5 (5·6)	0·597	60·7 (13·1)	0·466
	Married	59·7 (13·8)	0·011	59·2 (12·1)	0·170	46·6 (7·0)	0·345	51·0 (11·3)	0·562
	Widowed	55·4 (15·3)	0·717	46·0	0·362	NA	..	NA	..
	Divorced or separated	58·1 (14·0)	0·686	64·6 (9·3)	0·048	NA	..	NA	..
Number of births									
	1	58·6 (14·9)	Ref	60 (11·9)	Ref	45·9 (7·8)	Ref	55·0 (11·8)	Ref
	2	60·1 (14·1)	0·236	58·9 (13·0)	0·378	47·1 (5·9)	0·426	56·4 (11·6)	0·033
	3	60 (12·7)	0·302	62·2 (11·2)	0·150	46·2 (7·2)	0·822	55·8 (11·9)	0·321
	4 or more	57·8 (13·6)	0·523	62·7 (11·9)	0·168	46·5 (6·8)	0·628	56·2 (10·7)	0·157
Education									
	No school or primary	58·1 (14·1)	Ref	59·6 (12·0)	Ref	46·4 (6·5)	Ref	55·5 (11·7)	Ref
	Postprimary, vocational, or secondary	59·2 (13·9)	0·284	61·0 (12·3)	0·248	46·8 (8)	0·727	55·6 (11·7)	0·874
	College or above	62·1 (13·2)	0·007	59·6 (13·0)	0·991	43·3 (9)	0·449	57·1 (11·0)	0·052
Employed									
	No	57·4 (13·6)	Ref	61·1 (11·9)	Ref	46·5 (6·7)	Ref	55·6 (11·5)	Ref
	Yes	63·7 (14·1)	<0·0001	59·5 (12·6)	0·124	46·4 (9·2)	0·976	58·1 (13·2)	0·030
Wealth quintile									
	Poorest	56·4 (14·1)	Ref	NA	..	47·8 (8·2)	Ref	54·1 (10·7)	Ref
	Poor	57·8 (13·5)	0·341	56·0	Ref	45·3 (5·4)	0·056	55·6 (11·1)	0·071
	Middle	58·6 (14·5)	0·169	61·9 (12·7)	0·641	46·4 (6·5)	0·193	57·3 (11·3)	<0·0001
	Rich	61·7 (13·4)	<0·0001	58·3 (12·9)	0·851	48·3 (11·5)	..	56·2 (12·7)	0·008
	Richest	60·4 (14·1)	0·006	60·6 (12·1)	0·711	33	..	55·7 (12·2)	0·046
Delivery facility type									
	Government hospital	57·2 (13·9)	Ref	59·4 (12·2)	Ref	45·3 (10·5)	Ref	53·3 (11·8)	Ref
	Government health centre	59·3 (13·7)	0·036	63·8 (12·0)	0·001	46·5 (6·1)	0·494	60·4 (9·8)	<0·0001
	Mission or private facility	63·9 (14·1)	<0·0001	NA	..	46·7 (6·7)	0·391	NA	..
Delivery provider gender									
	Male	59·1 (13·5)	Ref	60·8 (11·7)	Ref	48·6 (8·8)	Ref	61·3 (10·8)	Ref
	Female	58·4 (14·1)	0·493	60·2 (12·3)	0·727	46·5 (6·8)	0·395	55·8 (11·6)	0·154
	Both	66·1 (14·8)	0·005	59·9 (12·8)	0·666	39·0 (8·5)	0·078	0·0 (0·0)	..
Pregnancy complications									
	No	58·2 (14·0)	Ref	60·3 (12·2)	Ref	45·4 (5·9)	Ref	56·7 (11·9)	Ref
	Yes	60·0 (13·9)	0·062	59·7 (12·7)	0·640	47·5 (7·7)	0·030	55·5 (11·5)	0·060

SD is missing when there was only one respondent for that group. NA=no observation under that category for the sample.

**Table 6:** Multivariable linear regression of person-centred maternity care score on selected predictors

		**Rural Kenya (n=850)**		**Urban Kenya (n=530)**		**Ghana (n=186)**		**India (n=2010)**	
		Coefficient (95 % CI)	p value	Coefficient (95 % CI)	p value	Coefficient (95 % CI)	p value	Coefficient (95 % CI)	p value
Age									
	15–19 years	0 (ref)	..	0 (ref)	..	0 (ref)	..	0 (ref)	..
	20–29 years	0·33 (−2·52 to 3·18)	0·820	2·38 (−2·76 to 7·52)	0·363	3·44 (−2·07 to 8·95)	0·219	0·84 (−1·75 to 3·43)	0·524
	30– 48 years	0·47 (−3·31 to 4·24)	0·809	4·36 (−1·71 to 10·4)	0·159	2·49 (−3·65 to 8·62)	0·425	2·09 (−0·93 to 5·12)	0·175
Currently married		2·54 (0·13 to 4·96)	0·039	−4·27^‡^ (−6·62 to −1·91)	<0·0001	1·23 (−4·01 to 6·46)	0·644	−0·83 (−7·33 to 5·67)	0·802
Number of births									
	1	0 (ref)	..	0 (ref)	..	0 (ref)	..	0 (ref)	..
	2	0·26 (−2·49 to 3·00)	0·855	−0·98 (−3·62 to 1·66)	0·466	−0·15 (−3·92 to 3·62)	0·937	0·86 (−0·36 to 2·08)	0·169
	3	−0·69 (−3·73 to 2·34)	0·653	2·28 (−1·26 to 5·82)	0·206	−0·46 (−4·49 to 3·57)	0·822	0·81 (−0·63 to 2·25)	0·272
	4 or more	−1·9 (−5·13 to 1·32)	0·247	2·3 (−2·66 to 7·26)	0·362	0·039 (−4·24 to 4·32)	0·986	0·47 (−1·32 to 2·27)	0·607
Education									
	No school or primary	0 (ref)	..	0 (ref)	..	0 (ref)	..	0 (ref)	..
	Postprimary, vocational, or secondary	0·38 (−1·80 to 2·57)	0·732	1·86 (−0·54 to 4·26)	0·128	0·89 (−1·97 to 3·75)	0·540	0·94 (−0·17 to 2·05)	0·098
	College or above	−0·23 (−3·64 to 3·19)	0·897	1·27 (−2·04 to 4·59)	0·450	−0·83 (−9·82 to 8·15)	0·855	2·11 (0·44 to 3·79)	0·013
Household wealth									
	Poorest or poor	0 (ref)	..	0 (ref)	..	0 (ref)	..	0 (ref)	..
	Middle	1·84 (−0·79 to 4·46)	0·170	3·1 (−21·4 to 27·6)	0·804	−2·31 (−5·06 to 0·44)	0·099	2·24 (0·90 to 3·57)	0·001
	Rich or richest	2·61 (0·37 to 4·84)	0·022	3·4 (−20·4 to 27·2)	0·779	−1·26 (−3·81 to 1·28)	0·330	1·83 (0·66 to 3·00)	0·002
Employed		5·21 (3·01 to 7·42)	<0·0001	−2·60 (−4·77 to −0·44)	0·019	1·1 (−2·64 to 4·85)	0·562	3·21 (1·09 to 5·33)	0·003
Facility type									
	Government hospital	0 (ref)	..	0 (ref)	..	0 (ref)	..	0 (ref)	..
	Government health centre	3·17 (1·21 to 5·12)	0·002	4·53 (1·78 to 7·28)	0·001	−0·0088 (−3·94 to 3·92)	0·996	7·27 (6·25 to 8·30)	<0·0001
	Mission or private facility	4·53 (1·68 to 7·37)	0·002	..	..	0·22 (−3·42 to 3·86)	0·906	..	..
Delivery provider gender									
	Male	0 (ref)	..	0 (ref)	..	0 (ref)	..	0 (ref)	..
	Female	−0·28 (−2·11 to 1·54)	0·761	−0·57 (−3·69 to 2·55)	0·720	−1·12 (−6·38 to 4·15)	0·677	−8·22 (−15·4 to −1·00)	0·026
	Both	6·72 (2·05 to 11·4)	0·005	−0·24 (−4·06 to 3·58)	0·901	−7·7 (−19·7 to 4·28)	0·206	..	..
Had pregnancy complications		1·69 (−0·13 to 3·51)	0·068	−0·83 (−3·67 to 2·00)	0·563	1·33 (−0·93 to 3·59)	0·246	−0·78 (−1·97 to 0·40)	0·195
Postpartum length ≥1 week		−6·81 (−10·0 to −3·62)	<0·0001	..	..	1·73 (−1·08 to 4·55)	0·225	..	..
Interviews in community		−2·56 (−4·43 to −0·69)	0·007	..	..	..	..	..	..
Constant		60·1 (55·8 to 64·3)	<0·0001	57·0 (32·7 to 81·3)	<0·0001	42·5 (34·0 to 51·0)	<0·0001	59·5 (49·5 to 69·4)	..

The sample sizes shown in the headers are lower than the full sample size for each group because we only used observations without missing data on person-centred maternity care and on the predictors in the model. The proportion of missing variables in each sample used for the multivariate analysis is 4·9% for rural Kenya, 0·2% for urban Kenya, 18·0% for Ghana, and 0·4% for India.

For the urban Kenya group, the only significant associations were in marital status and type of facility. Women who were cohabiting or divorced reported a higher PCMC score than did single or married women; and women who delivered in health centres reported a higher PCMC score than did those who delivered in hospitals. These associations remained significant in the multivariate analysis. In addition, the effect of employment became significant in the multivariate analysis, but not in the expected direction, with unemployed women reporting higher PCMC scores than employed women. In Ghana, only pregnancy complication is significant in the bivariate analysis and none of the associations were significant in the multivariate analysis.

In India, employed and wealthier women reported a higher PCMC score than did unemployed and poorer women, as did women who delivered in the health centres when compared with those who delivered in hospitals, in both the bivariate and multivariate analysis. In addition, the effect of education became significant in the multivariate analysis, with college educated women having a higher PCMC score than women with less than primary education. Also, the PCMC score appears to be higher with male than female providers, but this is an unstable estimate because only nine women reported being delivered by male providers in the India group.

## Discussion

To our knowledge, this is the first study to examine PCMC in more than one country with the same tool. Across four different study settings in three countries in sub-Saharan Africa and South Asia, we found that women are not getting person-centred care during childbirth in health facilities. The highest PCMC score across the four settings was about 60 (out of 90), with the domains of communication and autonomy scoring the lowest. It seemed that providers rarely introduced themselves, asked permission to do exams or procedures, explained procedures and purpose of medications to women, or created an environment in which women felt able to ask questions regarding their care. Women also had little involvement in decisions about their care, including the possibility of having birth companions present and the opportunity to choose their preferred delivery positions. Less than 5% of respondents reported physical abuse, and 11–18% reported verbal abuse—which is encouraging compared with previous studies^30^, ^39^ reporting much higher prevalence of physical and verbal abuse in some settings. However, PCMC entails more than the absence of abuse. Finally, we showed that PCMC differed by socioeconomic status in at least two settings and by level of facility in at least three settings. This study clearly indicates that much work is needed to improve the quality of maternity care that women receive when they deliver in health-care facilities throughout the developing world.

Previous studies on PCMC have focused on disrespect and abuse and have reported varying prevalence (15–98%), which is partly due to different methodological approaches.^24^ These discrepancies make it difficult to meaningfully compare our results with previous studies. Nonetheless, we found similar results to a study in Kenya^25^ that found the prevalence of physical abuse to be 4·2%. Other studies^27^, ^28^, ^38^ have documented a physical abuse prevalence of about 3%, similar to our findings for Ghana and India, whereas some studies^26^, ^30^, ^39^ have documented prevalence values above 30%. At the same time, the prevalence of non-consented care across five studies in sub-Saharan Africa ranged from less than 1% to 26%.^24^ Our findings suggest a higher proportion of non-consented care, particularly in India, where over 70% of women reported that providers never asked permission before examinations and procedures. Poor communication and lack of respect for women's autonomy has been documented in other studies in India.^27^, ^28^

We are able to compare the four groups in this study because they used the same data collection tool, although data collection approaches were different. Evidence from previous studies^26^, ^38^ suggests that women report better care when interviewed at the health facility and right after delivery. Our findings from the rural Kenya group are consistent with these studies: women reported lower PCMC when interviewed after a week of delivery and in their homes than when interviewed within a week of delivery and in health facilities. Thus, the different methodological approaches might explain some of the differences between different settings.

Population characteristics might also explain the differences across the settings. For example, PCMC is expected to vary by socioeconomic status. In fact, most of the women in the urban Kenya group, which had the highest PCMC score, were more educated and in the highest wealth groups, while most of the women in the Ghana group, which had the lowest score, had no education and were in the lowest wealth groups. This might explain the difference in the scores for these two settings. The homogeneity of these two groups might also explain why we did not find significant wealth and education differences in PCMC, as were found in the rural Kenya and India groups, which were more heterogeneous. The negative association with employment in the urban Kenya group might be due to the effect of employment being conditional on wealth;^34^ and the absence of significant associations in Ghana could be due to lack of power because of the much smaller group size than the groups in the other countries. Future applications of the PCMC tool in studies that use consistent methods across countries could allow for better cross-country comparisons.

There are other limitations to the study. First, the groups are not representative of the countries or the subregions data were collected in, as convenience sampling approaches were used. Second, the data are based on self-reports, thus are subject to social-desirability and recall biases. Furthermore, assessment of PCMC is complicated by women's potential fear of complaining about health-care providers or the treatment they received—especially when interviews take place in health-care facilities. Women might also not remember details of the encounters they had with providers during painful labour, and the recall of their experiences might be clouded by the outcome of their pregnancies—especially when interviews occur right after the delivery. Additionally, how women respond to questions on their experiences is affected by their expectations and by what is accepted as normal,^40^ such that mistreatment tends to be under-reported in interviews when compared with direct observations.^24^, ^28^, ^41^ This analysis might, therefore, be underestimating the true burden of poor PCMC in these settings.

The strength of our study is that we used a standard validated tool across diverse groups representing data from over 3000 women, who delivered in over 60 different facilities, in four separate locations, in three countries, across two continents. The multidimensional tool with both subjective and partly objective questions enables us to capture some of the contextual-level and individual-level factors that affect women's interpretation of their experiences, and to identify actionable targets for change. The subjective questions, such as whether the interviewee thinks she was treated with respect, allow women to respond on the basis of whatever respect means to them; however, these questions are less actionable because of the different meanings different people might associate with respect. Conversely, the partly objective questions (eg, whether a provider introduces herself or himself) represent standards of care that might or might not be important to every woman, but which provide actionable indicators for interventions. For example, about half of women in the India group felt respected all the time, although almost all the providers never introduced themselves, and less than 20% of them experienced verbal or physical abuse. Thus, the individual items by themselves might not reflect women's perceptions of PCMC, but together they approximate the complex construct of PCMC.

Our findings have several implications. First, PCMC is critically lacking in patient–provider interactions. For example, whether providers introduced themselves is rarely assessed in surveys of patient experiences. Qualitative work in Kenya,^35^ however, showed that women reported greater comfort with providers who introduced themselves. This simple act at the first encounter with health providers might, therefore, be a first step towards improving PCMC in LMICs. Second, not giving information to women about their care and not making them feel involved in it is compounded by their perception of not being able to ask questions. Simple questions from the provider to the patient (such as asking the patient in a meaningful way whether they have any questions and subsequently providing simple explanations) could make a big difference. Third, although overt disrespect and abuse is not prevalent, it is still reported as happening, which should be a cause for concern. Previous work^42^ suggested that some providers are abusive because of perceived lack of cooperation from patients. Therefore, interventions to help providers develop alternative ways of working with patients that they consider uncooperative are needed.

In addition, the finding that across the study settings PCMC scores tend to be higher in the low-level facilities than in the high-level facilities implies that increased effort is needed to improve PCMC in the high-level facilities. High-level facilities typically have better infrastructure and more skilled personnel than do low-level facilities and are, therefore, assumed to offer better clinical quality. However, the same assumption cannot be made for PCMC. Our qualitative work in these research settings suggests that women often do not like to be referred to high-level facilities because of fear of mistreatment (unpublished results). Efforts to improve PCMC in high-level facilities will therefore be crucial in reducing fear of referrals, which often leads to delays in the delivery of critical care for complications. Efforts towards addressing the socioeconomic status disparities are also critical.

This Article shows that across four different study settings in three countries in sub-Saharan Africa and South Asia, women are not receiving person-centred care during facility childbirth. While overt disrespect and abuse is not common, basic aspects of the patient–provider interaction are lacking, particularly in the area of communication and respect for women's autonomy. More efforts are, therefore, needed in LMICs to improve PCMC. Such efforts should include provider training on the importance of PCMC, patient and provider rights, and strategies to improve provider interactions with patients and their families. Providers should also be trained on how to appropriately handle conditions that often lead to poor interactions with women; for example, they should be taught coping mechanisms for stress and instructed on how to address biases that might affect how they provide care to some groups. Measurement and accountability mechanisms should also be implemented to reinforce efforts to improve PCMC, and these efforts should be in the context of broader health-systems strengthening.
